# Serum Tumor Necrosis Factor-Alpha as a Competent Biomarker for Evaluation of Disease Activity in Early Rheumatoid Arthritis

**DOI:** 10.7759/cureus.15314

**Published:** 2021-05-29

**Authors:** Mehreen Inam Illahi, Sofia Amjad, Syed Mehfooz Alam, Syed Tousif Ahmed, Murk Fatima, Moazzam A Shahid

**Affiliations:** 1 Physiology, Ziauddin University, Karachi, PAK; 2 Rheumatology, Liaquat National Hospital, Karachi, PAK; 3 Biochemistry, Ziauddin University, Karachi, PAK

**Keywords:** rheumatoid arthritis, autoimmune disease, erythrocyte sedimentation rate, disease activity score, tumor necrosis factor alpha

## Abstract

Aim

The cytokines particularly tumor necrosis factor-alpha (TNF-α) have a substantial role in the pathophysiology of rheumatoid arthritis (RA). The goal of this study was to evaluate the role of serum TNF-α as a competent biomarker of disease activity in RA and to assess the correlation of serum TNF-α with DAS28-ESR (disease activity score-erythrocyte sedimentation rate in 28 joints) and other markers expressed in serum of RA patients.

Methodology

The study was conducted from May 2020 to October 2020 after approval from the Ethical Review Committee of Ziauddin University. This cross-sectional study included 90 diagnosed cases of RA from 30 to 65 years with the complaint of arthralgia. Patients from the rheumatology clinic were enrolled in the study by a non-probability consecutive sampling technique. Informed consent was taken from each patient and they were assessed through a set of questions based upon disability in the performance of daily activities due to RA. Evaluation of serum levels of anti-cyclic citrullinated peptide (ACCP), rheumatoid factor (RF), erythrocyte sedimentation rate, and TNF-α were done by enzyme-linked immunosorbent assay (ELISA). Patients were segregated into groups based upon DAS28-ESR with erythrocyte sedimentation rate as an inflammatory marker. The Kruskal Wallis test was applied for the comparison of different variables in these groups. Spearman correlation was applied for the association between different variables. Multiple variable analysis was performed to assess the predictability of disease activity by serum markers included in the study.

Results

The results of our study disclosed a significant difference in ACCP, TNF-α, tender joint count of 28 joints (TJ-28), swollen joint count of 28 joints (SJ-28), and health assessment questionnaire-disability index (HAQ-DI) in disease activity groups. A significant correlation of serum TNF-α with DAS28-ESR in RA patients was observed.

Conclusion

This study illustrated a significant correlation of serum TNF-α with DAS28-ESR in RA patients. We found that expression of serum TNF-α may intensify the inflammatory activity in early RA, therefore, RA patients must be screened for this cytokine to monitor that disease activity could be useful for patients undergoing anti-TNF therapy.

## Introduction

Cytokines belong to a diverse family of proteins associated with the inflammatory activity in autoimmune diseases. They portray a significant role in the preservation of homeostasis. An imbalance in the cytokine network leads to enhanced inflammation. Therefore, these cytokines might be valuable as predictive biomarkers of disease activity [1,2]. Cytokines perform an essential role in the cascades that cause articular destruction and the comorbidities associated with immune-mediated joint diseases including rheumatoid arthritis (RA) [3]. RA is a chronic, systemic inflammatory, autoimmune disease that causes deformity and restriction of joint movements [4]. Cytokines are produced by innate immune cells of the synovial membrane in RA patients [3].

Among these cytokines tumor necrosis factor-alpha (TNF-α) is a principal cytokine, that is chiefly produced by macrophages [3]. TNF-α itself acts as a potent inducer of other proinflammatory cytokines and chemokines, thus further enhancing the inflammatory response [5]. In conjunction with other pro-inflammatory mediators, TNF-α promotes the stimulation of synovial fibroblasts, chondrocytes, and osteoclasts that release tissue-destroying enzymes, the matrix metalloproteinases (MMPs) [6,7]. The MMPs cause degradation of extracellular matrix components thus causing destruction of bone and cartilage which begins very early in the course of RA [8]. Hence, all of these TNF-α activities fuel inflammation in the synovium, increase angiogenesis, promote cartilage and bone resorption [3], suppress regulatory T cells, and promote pain [9,10]. TNF-α also amplifies osteoclast activation and differentiation [11]. According to a study, the severity of inflammation was assessed well in RA patients by TNF-α in both pre-treatment and post-treatment phases [7,12].

It has been proposed by many studies that disease activity can be monitored by diagnostic markers for RA, the rheumatoid factor (RF), and anti-cyclic citrullinated peptide (ACPP) [13,14]. However, the levels of these biomarkers do not correlate well with disease activity in RA. There is a growing need to search for new biomarkers to monitor the inflammatory activity in RA and in this context cytokines can be potentially applicative.

In RA, the inflammatory activity can be evaluated by a disease activity score with an erythrocyte sedimentation rate (ESR) as the inflammatory marker (DAS28-ESR) in 28 joints. Evaluation of disease activity guides the clinicians and patients toward a standardized treatment approach. The DAS28-ESR is a widely used measure for the clinical assessment of RA patients. It is a score that includes 28 tender and swollen joint counts, along with a patient global assessment, and a physician global assessment. Values are defined by high disease activity (HAD), moderate disease activity (MDA), low disease activity (LDA), or remission. Improvement or worsening of disease is reflected by changes in score values [15]. The goal of our study was to assess the efficacy of serum TNF-α as a potential biomarker of disease activity in early RA. We evaluated the correlation of serum TNF-α with DAS28-ESR and other markers expressed in serum of RA patients.

## Materials and methods

This cross-sectional study was conducted from May 2020 to October 2020 after approval from the Ethical Review Committee of Ziauddin University (Refcode: 0920319MIPHY) and included 90 diagnosed cases of RA from 30 to 65 years with the complaint of arthralgia. Patients from the rheumatology clinic were enrolled in this study on the basis of a non-probability consecutive sampling technique. Patients were diagnosed on the basis of 2010 American College of Rheumatology/European League Against Rheumatism (ACR/EULAR) criteria for RA [16]. Study participants were disease-modifying antirheumatic drugs (DMARD) naïve. All participants gave informed consent and filled in a questionnaire about the performance of their activities in daily life as assessed by the Stanford University Health Assessment Questionnaire-disability index (HAQ-DI) [17] which included 20 questions regarding, (1) dressing and grooming, (2) arising, (3) eating, (4) walking, (5) hygiene, (6) reach, (7) grip, and (8) common daily activities. The response of patients were recorded as scores from 0 to 3, mentioned in HAQ-DI: 0=able without any difficulty, 1=able with some difficulty, 2=able with much difficulty, and 3=unable. Clinical examinations were done by a certified trained rheumatologist. The total HAQ score was calculated as given in HAQ-DI. The DAS28 - including 28 tender (TJ28) and swollen joint (SJ28) count, and the ESR - was used to assess clinical disease activity by a DAS28-ESR calculator [18]. Values of DAS28-ESR were defined: HDA (>5.10), MDA (3.21-5.10), LDA (2.61-3.20), or remission (<2.60) [19].

A venous blood sample of 5 ml was taken from each patient by a trained phlebotomist and it was centrifuged at this 3000 rpm to get the serum and stored in multiple aliquots at −80 °C. RF levels were determined using an RF enzyme-linked immunosorbent assay (ELISA) kit (MBS721682; MyBioSource, San Diego, California, USA). The reference range was from 5.0 to 100 IU/mL, and analytical sensitivity was 1.0 IU/mL. According to the manufacturer’s protocol, the assay sample and buffer were incubated together with RF-horseradish peroxidase (RF-HRP) conjugate in a microtiter plate pre-coated with anti-RF antibody for one hour. The wells were washed and incubated with a substrate for the HRP enzyme. A blue-colored complex was formed and later after the addition of stop solution color changed to yellow. The intensity of color was measured spectrophotometrically at 450 nm in a microplate reader.

ACCP levels were determined using an ACCP antibody, ELISA kit (MBS720363; MyBioSource). The reference range was from 25 to 500 U/mL and analytical sensitivity of 1.0 U/ml. According to the manufacturer’s protocol, the assay sample and buffer were incubated together with CCP-Ab-HRP conjugate in CCP-Ab pre-coated plate for one hour. The wells were washed and incubated with a substrate for the HRP enzyme. A blue-colored complex of enzyme-substrate reaction was formed. A stop solution was added to terminate the reaction. The intensity of color was measured spectrophotometrically at 450 nm in a microplate reader.

Assay of serum TNF-α was done by a sandwich ELISA (Cat.No.E0082Hu; Bioassay Technology Laboratory, Shanghai, China). The reference range was from 3 pg/ml to 900 pg/ml and analytical sensitivity of 1.52 pg/ml. According to the manufacturer’s protocol, TNF-α present in the sample is added and binds to human TNF-α antibodies coated on the wells. Then, streptavidin-HRP is added and binds to the biotinylated TNF-α antibody. After incubation plate wells were washed. Substrate solution was added and color developed in proportion to the amount of human TNF-α. The reaction was terminated by the addition of stop solution and absorbance was measured at 450 nm.

Statistical analysis

Data analysis was done on Statistical Package for Social Sciences (SPSS), version 20 (IBM SPSS Statistics, Armonk, NY). Median and interquartile ranges were calculated for numeric variables The Kruskal Wallis test was applied for comparison of three groups. Spearman's correlation was applied for finding a correlation among the study variables. Multiple variable analysis was performed to assess the predictability of disease activity by serum markers included in the study.

## Results

Table 1 shows metabolic and biochemical variables among three groups of DAS28-ESR in RA patients. The Kruskal Wallis test was applied. Significantly (p≤0.001) higher levels of serum ACCP were seen in the severe DAS28 group, versus the mild and moderate groups. The TJ-28 was significantly (p≤0.001) high in mild and severe DAS-28 groups when compared with the moderate group. A significantly high SJ-28 (p≤0.001) count was seen in the severe DAS28 group than in the mild and moderate groups. The HAQ-DI was significantly (p=0.008) high in the mild disease activity group than in the severe and moderate sub-groups. The serum TNF-α was significantly (p≤0.001) high in severe DAS28 scores versus the moderate and mild disease score groups. Among the three groups of DAS28-ESR, no significant difference was noted in BMI, RF, and ESR values.

**Table 1:** Comparison of metabolic, biochemical, and clinical parameters in groups of DAS28-ESR. *≤0.05 p-value. **≤0.001 p-value. BMI: body mass index; DAS28-ESR: disease activity score of 28 joints; RF: rheumatoid factor; ACCP: anti-cyclic citrullinated peptide; ESR: erythrocyte sedimentation rate; TNF-α: tumor necrosis factor-alpha; TJ-28: tender joint count of 28 joints; SJ-28: swollen joint count of 28 joints; HAQ-DI: health assessment questionnaire disability index.

Parameters	DAS28-ESR mild (2.61–3.2)		DAS28-ESR moderate (3.21–5.1)		DAS28-ESR severe (>5.1)		p-Value
	M	IQR	M	IQR	M	IQR	
BMI (kg/m²)	21.6	2.6	23	3.07	22.4	2.3	0.101
RF (IU/L)	15	133	12	66	38.3	125	0.387
ACCP (U/L)	97	198.2	10	99	154	0.00	0.007*
ESR (mm/hr)	21	16	35	20	39	55	0.076
TNF-α (pg/ml)	59	22.59	164.8	60.2	254.0	104.6	
TJ-28	12	19	6.00	3.75	12	5.00	
SJ-28	8.00	20	3.00	2.00	9.00	20	
HAQ-DI	1.13	0.95	0.63	0.75	1.00	0.75	0.008*

Our results regarding correlation of DAS28-ESR and serum TNF-α with biochemical, clinical, and metabolic parameters, showed significant correlation with serum ACCP (r=0.278, p=0.008), ESR (r=0.306, p=0.003), TJ-28 (r=0.415, p≤0.001, SJ-28 (r=0.413, p≤0.001), and serum TNF-α (r=0.533, p≤0.001). There was no correlation of BMI (r=0.048, p=0.655), RF (r=0.09, p=0.400), and HAQ-DI (r=0.194, p=0.67) with DAS28-ESR. Significant correlations of serum TNF-α with BMI (r=0.244, p=0.02), and DAS28-ESR (r=0.533, p≤0.001) was observed. There was no correlation of TNF-α with RF, ACCP, ESR, TJ28, SJ28, and HAQ-DI (Table 2).

**Table 2:** Correlation of DAS28-ESR and serum TNF-α with other variables. *≤0.05 p-value. **≤0.001 p-value. BMI: body mass index; DAS28-ESR: disease activity score of 28 joints; RF: rheumatoid factor; ACCP: anti-cyclic citrullinated peptide; ESR: erythrocyte sedimentation rate; TNF-α: tumor necrosis factor-alpha; TJ-28: tender joint count of 28 joints; SJ-28: swollen joint count of 28 joints; HAQ-DI: health assessment questionnaire disability index.

Clinical, metabolic, and biomarker parameters	DAS28-ESR r(p)	TNF-α
BMI (kg/m²)	0.048 (0.655)	0.244 (0.020*)
RF (IU/L)	0.090 (0.400)	−0.109 (0.306)
ACCP (U/L)	0.278 (0.008*)	−0.027 (0.804)
ESR (mm/hr)	0.306 (0.003*)	0.018 (0.864)
TNF-α (pg/ml)	0.533 (≤0.001)	1.00
TJ-28	0.415 (≤0.001)	0.122 (0.254)
SJ-28	0.413 (≤0.001)	0.198 (0.061)
DAS28-ESR	1.00	0.533 (≤0.001)
HAQ-DI	0.194 (0.067)	0.005 (0.964)

Multiple variable analysis was performed based upon the results of Spearman’s correlation coefficient analysis. Our results showed no significant association between DAS28 and RF, ACCP, and ESR. Moreover, with every one‐unit increase in serum TNF-α, chances of disease activity will increase by 9.6% (Table 3).

**Table 3:** Multiple variable analysis with disease activity (DAS28-ESR).

Outcome variable	Predictor variables	β	Wald	Adjusted prevalence ratio	95% CI	p-Value
DAS28	RF	0.015	2.56	1.015	0.997–1.034	0.109
	ACCP	2.00	2.14	7.42	0.507–108.51	0.143
	ESR	0.026	0.018	1.027	0.991–1.064	0.145
	TNF-α	0.096	8.365	1.101	1.031–1.175	0.004*

## Discussion

In our study, serum TNF-α was found to be significantly associated with DAS28-ESR in early RA patients. Patients with a low disease activity score had a significantly lower concentration of serum TNF-α than in the subgroups with moderate and high disease activity. Similar to our findings were the observations made by other studies conducted on newly diagnosed RA patients [5,7,19]. These findings show that TNF-α is expressed earlier in the pathologic events in DMARD naïve RA patients and can therefore be an important marker for the evaluation of disease activity. Previously in a study patients with high disease activity scores were reported to have significantly high concentrations of TNF-α in blood and synovial fluid as well [20]. However, in our study, synovial fluid samples were not taken.

The ACCP levels were higher in our study participants with severe DAS28 scores. This is maybe due to the high inflammatory activity in the majority of the patients positive for ACCP [21]. The values of TJ-28, the SJ-28, and HAQ-DI were higher in patients with severe disease activity. Associations of these variables with disease activity have also been reported in a previous study [22]. The reason for this can be the release of cytokines earlier in the course of RA that results in joint inflammation and pain, hence altering the functional ability in these patients [21]. BMI, RF, and ESR values showed no significant differences in the three groups of DAS28-ESR.

Serum TNF-α was shown to have a positive correlation with DAS28 in our study participants. Previous studies also showed a positive correlation between plasma levels of TNF-α and activity of the disease in the newly diagnosed RA patients and not in patients who were on DMARD treatment [7,19]. Shrivastava et al. and others reported that DAS28 scores showed a significant correlation with serum TNF-α in the high disease activity subgroup of RA patients [7,23,24]. These findings suggest that TNF-α is a substantial mediator of inflammation in RA and plays a key role in the development and advancement of RA. However, Gheita et al. reported a significant negative correlation of the serum TNF-α level with the DAS28 in one genotype group and association between these two variables in the other genotype group of their study participants. A possible explanation for the varied associations between disease activity and serum TNF-α levels may be due to the TNF-α gene promoter polymorphism [25]. Our study showed a positive correlation of TNF-α with BMI. Similar findings were shown in a previous study [26]. The association between BMI with inflammation may be due to the increased levels of adipocytokines that act upon innate immune cells thus activating monocytes that produce increased levels of TNF-α [27]. Our study did not find any correlation of serum TNF-α levels with tender joint count and ESR in contrast to the association between TNF-α levels with TJ-28 and ESR in RA patients reported by Yen et al. [28].

A significant correlation of DAS28 with serum ACCP was also observed. The ACCP-containing immune complexes (ACCP-IC) triggers the release of inflammatory cytokine TNFα via the Fcγ R-dependent pathway by macrophages [29]. These cytokines attack the synovium in joints of RA patients and lead to aggressive disease and ultimately results in joint erosion [21], hence, there is a more inflammatory activity in ACCP positive patients. Our study did not show a correlation between TNF-α and ACCP and RF titers. However, these variables correlated slightly but significantly in other studies [30].

Multivariable analyses revealed that high levels of serum TNF-α shows an increased probability of severity in disease activity in RA patients. However, Dissanayake et al. in their study observed a significant association of DAS28 with the cellular expression of TNF-α [4].

This research highlights not only the importance of TNF-α alpha in the severity of disease in early RA patients but can also contribute to the monitoring of disease activity patients on DMARD treatment. There were certain limitations to our study. It was a single-centered cross-sectional study and patients’ samples were collected only once. They were not followed up after few months of treatment for RA. To validate our results, we propose that, multicenter studies, with a larger sample size and patient progress over a couple of months, are needed in selected RA patient groups.

## Conclusions

Our study has found a significant correlation of TNF-α with DAS28-ESR in newly diagnosed RA patients. The available therapeutic agents that target this cytokine have excellent clinical effects in RA patients. In future studies on targeting TNF-α in the early stage of RA and its evaluation to monitor the progress of disease could be useful for patients undergoing anti-TNF therapy. If these patients are followed up properly, then serum TNF-α levels together with the assessment of clinical activity of disease by DAS28 could be used as a guide in determining the dose and intervals between dosing of TNF-α inhibitors in order to accomplish the desired therapeutic response. The number of patients in our study was relatively small, however, our findings are highly suggestive and require further large-scale investigations in the future to make use of serum TNF-α levels as a potential biomarker for evaluation of disease activity both before and after the start of anti-TNF therapy.
